# Children and young people’s reported contact with professional services for mental health concerns: a secondary data analysis

**DOI:** 10.1007/s00787-023-02328-z

**Published:** 2024-01-04

**Authors:** Frances Mathews, Tamsin Jane Ford, Simon White, Obioha Chukwunyere Ukoumunne, Tamsin Newlove-Delgado

**Affiliations:** 1 University of Exeter Medical School, Exeter, UK; 2 Department of Psychiatry, School of Clinical Medicine, University of Cambridge, Cambridge, UK; 3 MRC Biostatistics Unit, University of Cambridge, Cambridge, UK; 4 Department of Health and Community Sciences, Faculty of Health and Life Sciences, NIHR Applied Research Collaboration South West Peninsula, University of Exeter, Exeter, UK

**Keywords:** Children, Young people, Mental health, Services, Secondary data analysis, MHCYP

## Abstract

Children and young people’s mental health services have been under increasing pressure following COVID-19. Understanding, for which channels help is sought from, will highlight services needing support. This study aims to explore the professional services that parents of children, and young people get help from when they have a concern for the child’s/their mental health. Secondary analysis of data is taken from Mental Health of Children and Young People in England Survey, 2017. 7608 reports of mental health-related contact with professional services from parents of 5–16 year-olds and self-reports from young people aged 17–19 were available. Service contact was reported by Diagnostic and Statistical Manual of Mental Disorders (DSM-V) diagnosis, age, gender and ethnicity. Less than two-thirds of children and young people with a DSM-V diagnosis (63.5% (95% CI 58.6–68.1) aged 5–10, and 64.0% (95% CI 59.4–68.4) aged 11–16) reported contact with any professional services. The figure was lower for those aged 17–19; 50.1% (95% CI 42.8–58.2), *p* = 0.005. Children and young people aged 5–16 from Black (11.7%; 95% CI 2.4–41.4), Asian (55.1%; 95% CI 34.7–73.9) and Mixed (46.0%; 95% CI 32.4–60.3) ethnic groups reported less contact with professional services compared to those from the White group (66.9%; 95% CI 63.5–70.2). Patterns of service access during the three main educational stages aid with understanding service need during childhood. These lower levels of reported service access for young people aged 17–19 with a DSM-V diagnosis and those in ethnic minority groups demand further investigation.

**Supplementary Information:**

The online version contains supplementary material available at 10.1007/s00787-023-02328-z.

## Introduction

With over half of all psychiatric disorders developing before the mid-teens, timely access to support and treatment is essential for prevention and early intervention [1–3]. Historically, prevention and treatment services for child and adolescent mental health have received disproportionately low levels of funding and resource, with UK-based clinical commissioning group reports in 2020/2021 showing an average spend of less than 1% of total budget [4]. This likely contributes to the substantial levels of unmet need, even in high-income countries [5].

Increasing policy focus and awareness around child mental health in many countries has been accompanied by rises in demand for services. In the UK, prior to the pandemic, there had been a longstanding trend of escalating referrals to child and adolescent mental health services (CAMHS) and rises in mental health-related emergency attendances, mirrored by increased mental health-related contacts with education professionals [6–8]. The extent to which these trends reflect increases in prevalence has been debated. Whilst data from the national surveys in England between 1999 and 2017 suggested a gradual rise in overall prevalence, particularly of emotional disorders, the influences of increased mental health awareness, changes in help-seeking behaviours including the increase of social media use, and interpretations of psychological distress may also play a role [9–13, 41]. Studies on the experience of access to services for those in need, however, repeatedly report barriers including lack of information, high thresholds for access, unclear referral criteria, and lack of specialist skills needed to accurately identify mental health problems [14]. Even those who access specialist mental health services can experience long waiting times for treatment and may not receive evidence-based interventions [15].

By definition, data on referrals and service contact only tell us about those who are seeking help from services. Only population surveys are able to report on those with psychopathology who are not already in contact with services and provide a broad picture of unmet need. Until recently, more up-to-date epidemiological data to assess trends in psychopathology and service contact on a population level have been lacking, resulting in a gap in knowledge about trends in service contact in relation to need, and access inequalities.

We present a secondary analysis of data from the Mental Health of Children and Young People in England (MHCYP), 2017 survey describing mental health-related service contact amongst a nationally representative probability sample [13]. As the data have only recently been made available for analysis in more depth, this paper provides the most recent pre-pandemic baseline measure of reported service use, representing a basis for measuring changes over the course of the pandemic. Our aim is to describe reported contact with a range of professional services for children and young people with mental health concerns for those meeting/not meeting criteria for a psychiatric diagnosis, by: (1) age group; (2) gender; (3) ethnicity; and (4) type of disorder.

## Methods

### Study population

Of the 18,029 parents of children and young people aged 2 to 19 who were invited to participate in the MHCYP (2017), 9117 participants completed survey responses (for further information please refer to ‘Survey Design and Methods Report’ [16]). This secondary analysis used responses from, 7654 parents of children, and young people aged 5–19 years.

### Procedure

MHCYP 2017 is a nationally representative cross-sectional study of children and young people aged 2–19 years, commissioned by National Health Service (NHS) Digital [13]. The National Health Service (NHS) Patient Register sampled eligible children and young people to ensure a representative selection of the population in England was contacted and participated in the survey. Primary informants were parents of children aged 2–16 years, and young people aged 17–19 years. Children aged 11–16 years were invited to complete their own version of the survey. Teachers of included 5- to 16-year-olds nominated by parents who they felt knew the child well were also invited to participate in the survey.

### Measures

The key measures used in our secondary analysis are described below:

### Measures of mental health and psychiatric diagnosis

Parents of children aged 5–16, young people aged 11–19 and, if the family agreed, teachers of those aged 5–16 completed the Development and Wellbeing Assessment (DAWBA), a structured diagnostic assessment combining highly structured questions with further semi-structured probes to elicit more detail when difficulties had been experienced. A team of trained clinical raters (including TND and TF) assessed question responses and free text from all informants, and assigned a DSM-V and ICD-10 diagnosis to relevant cases [17, 18].

### Measures of mental health-related service contact

Participants for children aged 5–16 years and young people aged 17–19 years were given the option to report contact with a range of informal support and professional services as detailed in Table 1.

**Table 1:** Service contact question and response categories

Question	‘In the past year have you or <Name> been in contact with any of these people because of worries about <your/his/her> emotions, behaviour, concentration or difficulties in getting along with people?’
Response categories	Example of professional’s role within category
Primary health care (PHC)	A GP, family doctor, health visitor, practice nurse or school nurse
Teachers and school staff (TSS)	A tutor, head of year, head teacher or special educational needs coordinator
Education specialist (ES)	Educational psychologist, educational social worker or specialist teacher from outside school
Mental health specialist (MHS)	A mental health nurse, psychiatrist, psychologist or counsellor
Physical health specialist (PHS)	A hospital or community paediatrician, or occupational therapist
Social care	A social worker
Youth justice	A probation officer or someone working in a Youth Offending Team

‘Informal’ service categories not being reported here were also available included: family or close friend; telephone helpline; self-help group; internet

### Socio-demographic characteristics

Other data collected at baseline and used in this analysis included: age, ethnicity, gender (see Survey Design and Methods Report [16]). The ethnicity categories were White (White British/Other), Black (Black/African/Caribbean/Black British), Asian (Asian/Asian British) and Mixed (Mixed/Multiple/Other). Age was split into three categories based on educational stage (5–10; 11–16 and 17–19 years), and gender was split by male and female (the only categories available).

### Secondary data analysis

STATA version 17.0 was used to undertake the analyses [19].

Psychiatric disorder was defined as any DSM-V diagnosis according to the DAWBA (any diagnosis/no diagnosis) as well as using broad categories of child psychiatric disorder [any anxiety disorder, any depressive disorder, any behavioural disorder, Attention-Deficit Hyperactivity Disorder (ADHD), Autism Spectrum Disorder (ASD)]. Comorbidity described those with two or more DSM disorders at this broad group level) versus those with one DSM disorder or no disorder).

We examined service contact in two ways. First, we reported any professional service contact (defined as contact with any of the professional services listed in Table 1) and a breakdown of key types of service, including TSS, PHC, ES, MHS and PHS. Second, we reported contact with any professional service and the most commonly reported services which included TSS, PHC and MHS.

Complete data on professional service contact were available on 99.4% (*n* = 7608) of the sample. As we report service contact prevalence, calibration weights were used to adjust the sample back to the population from which it was selected. Further information on MHCYP in 2017 survey weighting is available in the survey design and methods report [16].

The prevalence of contact with services is reported by DSM diagnosis status and by ethnic group category with 95% confidence intervals. Logistic regression was used to compare contact between these subgroups, reporting odds ratios (ORs) and 95% confidence intervals. Reporting of prevalence and logistic regression analyses were stratified by age group to reflect different stages of education (5–10 years, 11–16 years and 17–19 years). Ethnicity was split into four groups for the children aged 5–16 years (White; Black; Asian; Mixed), and two groups (White; Black, Asian and Mixed) for the 17–19-year-olds to account for small numbers. In reporting our findings, we followed the ONS and UK Data Service Statistical Disclosure Controls guidance, including rounding up the total number of individuals in the sample (N) to the nearest 5 [20].

## Results

This sample consisted of *n* = 7608 parents of children, and young people aged 5–19 years (mean age 11.2), with *n* = 3784 (49.7%) female. The majority of participants were of White ethnicity *n* = 6053 (79.6%), whilst 312 (4.1%) were of Black ethnicity, 774 (10.2%) were Asian and 467 (6.1%) were of Mixed ethnicity. Supplementary Table 1 summarises the unweighted characteristics of children and young people who reported contact with a professional for a mental health concern. Overall sample prevalence included 20.5% (19.2–21.8) of children aged 5–10, 21.5% (20.1–23.0) of those aged 11–16 and 22.0% (19.5–24.8) of those aged 17–19, who reported contact with any professional services.

Table 2 shows the weighted prevalence of contact with professional services for children and young people with or without a DSM-V diagnosis, by age group. Across any professional service, children and young people aged 5–10 and 11–16 with a DSM-V diagnosis were more likely to report access 63.5%, (58.6–68.1) and 64.0% (59.4–68.4) respectively. For young people aged 17–19 years, this figure was just over half: 50.5% (42.7–58.2). Access was not confined to those with a disorder, as children aged 5–10 and 11–16 years without a diagnosis also reported more frequent contact with TSS and PHC compared to specialist mental health services, indicating their gate keeping role.

**Table 2:** Weighted prevalence and odds ratios of those with and without a DSM-V diagnosis by age category 5–10 years, 11–16 years and 17–19 years reporting contact with professional services

Service type	Age group:	Age 5–10 years		Age 11–16		Age 17–19	
	DSM-IV diagnosis:	No diagnosis	Diagnosis	No diagnosis	Diagnosis	No diagnosis	Diagnosis
	*N*:	3155	425	2605	495	750	180
Any professional service	Percentage (95% CI)	14.2 (13–15.5)	63.5 (58.6–68.1)	13.2 (11.9–14.6)	64.0 (59.4–68.4)	15.6 (13.1–18.6)	50.5 (42.7–58.2)
	Unadjusted OR (95% CI)	Reference	10.2 (8.2–12.7)	Reference	12.3 (9.9–15.3)	Reference	5.5 (3.8–8.0)
Teacher and school staff	Percentage (95% CI)	12.0 (10.8–13.2)	52.8 (47.8–57.7)	10.3 (9.1–11.5)	50 (45.4–54.6)	16.7 (13.5–20.6)	25.6 (19.3–33.0)
	Unadjusted OR (95% CI)	Reference	8.0 (6.4–9.9)	Reference	9.0 (7.2–11.1)	Reference	3.0 (2.0–4.6)
Mental health specialist	Percentage (95% CI)	0.9 (0.6–1.2)	15.4 (12.2–19.4)	1.8 (1.3–2.5)	23.9 (20.3–28.1)	3.2 (2.1–4.8)	25.3 (19.3–32.4)
	Unadjusted OR (95% CI)	Reference	19.8 (12.6–31.0)	Reference	17.4 (12.2–24.8)	Reference	10.7 (6.4–18.0)
Primary health care	Percentage (95% CI)	3.3 (2.7–3.9)	33.5 (29.0–38.3)	2.8 (2.2–3.6)	29.7 (25.7–34.1)	5.5 (4.0–7.6)	22.8 (17.1–29.7)
	Unadjusted OR (95% CI)	Reference	14.6 (11.1–19.2)	Reference	14.8 (10.9–20.0)	Reference	6.0 (3.8–9.5)
Educational specialist	Percentage (95% CI)	1.7 (1.3–2.2)	25.8 (21.7–30.4)	1.7 (1.2–2.2)	22.9 (19.3–27.0)	1.8 (1.0–3.1)	7.4 (4.1–12.9)
	Unadjusted OR (95% CI)	Reference	18.8 (13.3–26.5)	Reference	17 (11.8–24.4)	Reference	4.1 (1.8–9.1)
Physical health specialist	Percentage (95% CI)	1.2 (0.9–1.7)	22 (18.1–26.3)	0.8 (0.5–1.3)	14.3 (11.4–17.9)	0.4 (0.1–1.4)	2.0 (0.6–6.2)
	Unadjusted OR (95% CI)	Reference	14.6 (11.0–19.2)	Reference	14.8 (10.9–20.0)	Reference	6.0 (3.8–9.5)

*OR* odds ratio

**TSS was the most commonly accessed service **by children with a DSM-V diagnosis, reported by 52.8% (47.8–57.7) of 5–10-year-olds, and 50.0% (45.4–54.6) of those aged 11 to 16. Contact reported with TSS was less common amongst 17–19 years, 25.6% (19.3–33.0) who reported similar prevalence of contact with MHS 25.3% (19.3–32.4) and PHC 22.8%, (17.1–29.7).

A third of those aged 5–10 and 11–16 years, and almost half of those aged 17–19 years with a DSM-V diagnosis reported ‘no’ service contact. For those without a DSM-V diagnosis, levels of contact for those aged 17–19 with TSS were higher than other age groups 16.7% (13.5–20.6). Similarly, the level of contact with PHC was higher for those aged 17–19 without a disorder [5.5% (4.0–7.6)] than those in other age groups.

The prevalence of reported contact with a MHS for children aged 5–10 with a DSM-V diagnosis was 15.4% (12.2–19.4), rising to just under a quarter for those aged 11–16 [23.9% (20.3–28.1)] and those aged 17–19 years [25.3% (19.3–32.4)]. Prevalence of reported contact with PHC for those with a DSM-V diagnosis compared to those without was higher than those aged 5–10, 33.5% (29.0–38.3) and those aged 11–16 years, 29.7%, (25.7–34.1). Whilst MHS, PHC, ES and PHS contact prevalence remains low across all age groups, children aged 5–10 and 11–16 with a DSM-V diagnosis are more likely to report contact with ES and PHS.

Table 3 shows the prevalence and odds ratios with 95% confidence intervals and overall prevalence *p*-value for children and young people aged 5–16 by four group ethnicity, and those aged 17–19 by two group ethnicity reporting contact with any professional services and the other most commonly reported services for those with DSM-V diagnosis.

**Table 3:** Prevalence and odds ratios with 95% confidence intervals of those with a DSM-V diagnosis by ethnicity, amongst children aged 5–16 and 17–19 reporting contact with professional services

Service type	Ethnic group	White British/other	Black/African/Caribbean/Black British	Asian/Asian British	Mixed/multiple/other	White	Black/Asian/mixed/other
	Age:	5–16 years				17–19 years	
	*N*:	825	15	25	55	145	35
Any professional service	Percentage (95% CI)	66.9 (63.5–70.2)	11.7 (2.4–41.4)	55.1 (34.7–73.9)	46 (32.4–60.3)	55.4 (44.7–61.8)	40.4 (24.5–58.7)
	Unadjusted OR (95% CI)	Reference	0.29 (0.2–0.5)	0.42 (0.3–0.6)	0.54 (0.4–0.8)	Reference	0.99 (0.6–1.6)
Teacher and school staff	Percentage (95% CI)	54.6 (51.1–58.1)	4.9 (0.5–33.3)	28.8 (14.0–50.2)	36.5 (24.0–51.1)	26.5 (19.5–35.0)	22.2 (10.5–40.9)
	Unadjusted OR (95% CI)	Reference	0.21 (0.1–0.4)	0.32 (0.2–0.5)	0.55 (0.4–0.8)	Reference	1.09 (0.6–1.9)
Mental health specialist	Percentage (95% CI)	20.5 (17.8–23.6)	11.7 (2.4–41.4)	17.9 (7.1–38.5)	17.6 (9.1–31.3)	27.9 (20.9–36.2)	15.8 (7.0–31.9)
	Unadjusted OR (95% CI)	Reference	0.35 (0.1–1.1)	0.57 (0.3–1.1)	0.57 (0.3–1.1)	Reference	0.61 (0.3–1.3)
Primary health care	Percentage (95% CI)	33.5 (30.2–36.9)	4.9 (0.5–33.3)	18.7 (7.7–38.9)	21.3 (11.8–35.4)	23.8 (17.4–31.6)	19.2 (8.7–37.3)
	Unadjusted OR (95% CI)	Reference	0.32 (0.1–0.8)	0.57 (0.3–0.9)	0.49 (0.3–0.8)	Reference	0.93 (0.5–1.8)

*OR* odds ratio

Overall, reported contact with any professional service for children and young people with a disorder aged 5–16 of Black ethnicity [11.7% (2.4–41.4)] and of Mixed ethnicity (46%; 95% CI 32.4–60.3) were significantly lower than those from a White ethnic background (66%; 95% CI 63.5–70.2). Those in the Black, Asian and Mixed ethnic groups were significantly less likely to report contact with TSS (4.9%, (0.5–33.3), 28.8%, (14.0–50.2) and 36.5%, (24.0–51.1), respectively) than those of White ethnicity [54.6%; (51.1–58.1)]. However, there were wide confidence intervals around estimates for the Black, Asian and Mixed ethnic groups across all service categories.

As with the younger age group, those aged 17–19 of White ethnicity reported highest levels of contact with any professional services, as well as TSS, MHS and PHC individually.

Supplementary Table 2a, b and c show contact with professional services for those with a DSM-V diagnosis aged 5–10 and 11–16 years as reported by parents and 17–19-year self-reports, by gender and mental health disorder status. Throughout the tables, similar levels of contact are seen with PS, TSS, PHS, and MHS for both boys and girls aged 5–10 and 11–16. For those aged 17–19, both boys and girls report similar levels of contact with professional services. In this age group, some figures were too small to report.

## Discussion

This is the first secondary data analysis to examine the prevalence of mental health-related service contact amongst children and young people with a DSM-V disorder in England using the most recent available national survey [13]. Our findings suggest most children and young people with a DSM-V diagnosis do not access mental health specialists. As in previous surveys, teachers and school staff were the most commonly reported professional service [10, 21]. Children aged 5–16 in Black, Asian and Mixed ethnic groups, had markedly lower levels of contact with any professional service than those from a White ethnic background. Overall, very few without a DSM-V diagnosis reported contact with professional services.

Less than one-fifth of those aged 5–10 years, and only slightly higher proportions of children aged 11–16 and young people aged 17–19 reported contact with a mental health specialist. This appears lower than estimates from other high-resourced countries, for example the School Children Mental Health in Europe Project found that just under a third of those with a disorder had at least one visit with a mental health professional in the past 12 months [5], although systems may not be directly comparable. There also appears to have been no increase in the proportion of those with a disorder in contact with specialist mental health services since the previous national survey in 2004 [10, 13]. It is important to note, however, that reported specialist mental health service contact does not necessarily equate to receiving evidence-based treatments, or in some cases, any treatment at all [4, 22]. Sawyer et al. [23] reported that only 11.6% of children in Australia with a disorder received ‘minimally adequate treatment’, defined as post-diagnosis completion of at least 8 visits with a mental health professional, or 4–7 visits plus medication. It is also possible that ‘adequate’ treatment is received from other professional service settings, for example school staff.

The prevalence of reports of contact with teachers and school staff for those with a DSM-V diagnosis remains at a very similar level to the previous BCAMHS survey in 2004 [10]. Schools have played an increasingly significant role in recognition and management of child mental health concerns in England, and following the Five Year Forward View [24] are embedding more specialist mental health teams within schools and colleges.

Our results replicate ethnic inequalities of those reporting contact with professionals [25]. Whilst our sample size resulted in low-precision estimates, they suggest that children from Black, Asian and Mixed ethnic groups were less likely to have mental health-related contact with teachers and school staff. Schools are key settings for identification and referral of problems, which may result in reduced access to support and treatment for these groups via this route. A similar trend was seen for young people, although differences between ethnic groups were not statistically significant, almost certainly reflecting lack of statistical power. A recent scoping review explored factors of those in ethnic minority groups which may explain differences in mental health-related help-seeking and service access, describing a lack of information, lack of trust in care professionals and cultural-based perceptions of mental resilience as potential barriers [26]. Maddock et al.’s [27] UK-based longitudinal studies during COVID-19, reported that those in ethnic minorities (excluding White minorities) were more likely to report healthcare disruption. Bains and Gutman [28] highlighted higher levels of internalising problems for those in ethnic minority groups which may contribute to reduced service access as such difficulties are harder to recognise. It is also fundamental that opportunities for structural systems to implement and improve strategies aimed at reducing ethnic-based bias and inclusive approaches are adopted to ensure access to mental health support for all [42].

In general, we found that young people were less likely to report contact with professional services than parents of children. This may relate to difficulties reported in other literature with transitioning to adult-based services, with increased risk of disengagement and parental support to navigate the system [29, 30]. Young people may experience higher levels of stigmatisation particularly where there are lower mental health resources which likely impacts their help-seeking [5, 14]. They also report the disparity between being heavily encouraged to seek help via advertisements for example posters in schools and the accessibility of support once they have reached out [31]. Another explanation is that ‘older’ young people develop their own help-seeking behaviours which include use of social media to inform and access information on mental health and their services, and contact with other ‘informal’ sources of support, which is outside the scope of this paper [14, 41].

## Strengths and limitations

This study benefitted from a large community sample carefully selected with analysis weighted to be representative of children and young people aged 5–19 in England. This allows us to examine naturalistic contact with the full range of services amongst the population of those meeting criteria for a diagnosis. The survey also used the same validated standardised diagnostic assessment used in the previous two BCAMHS, allowing a broad comparison of service contact levels between the three surveys [10, 11, 13].

We are mindful that the dataset imposes some limitations, for example, we were unable to examine service contact in relation to different gender identities, as there were only two reporting options (male/female). Despite the large population sample, small numbers of those from Black, Asian and Mixed ethnic backgrounds, particularly amongst those in the 17–19 age group, meant imprecise estimates of the percentage with contact. Similarly, amongst this age group, small numbers of those with certain diagnoses resulted in wide confidence intervals or unreportable results, which limited our ability to examine patterns of contact in detail. For 5- to 16-year-olds, we note that parents may not have always been aware of all services accessed by their child, and young people may consider reports of contact, particularly with teachers and school staff, differently to parents as they have more regular contact with them. We also note that reports of contact may not equate to evidence-based treatment. Finally, we present descriptive analyses; acknowledging that various individual, family and socio-economic factors will affect the relationships between demographics and service contact.

## Implications for policy, practice and research

This paper represents a baseline to examine how patterns of contact and of unmet need may have changed during the pandemic. Rising and sustained rates of probable mental health disorder for children and young people surveyed throughout the pandemic are likely to reflect an increased need for support [13, 32, 33]. COVID-19 restrictions in many settings limited accessibility of mental health support and saw the majority of provision move online, which is not necessarily suitable or available for all [4, 34, 35]. This is also likely to exacerbate the number of those electing not to seek help from services by parents and young people in a qualitative study by Mathews et al. [31]. Post COVID-19 restrictions, McNicholas et al. [36] found a sustained rise in referrals to CAMHS indicating a backlog of unsupported need. Community-based services were also providing increasing levels of low-intensity interventions [37].

Restrictions on school access during lockdowns and limited face-to-face learning also means there may have been delays in recognition and treatment of mental health problems that would usually have been identified and supported within a school setting [38, 39]. The current backlog of those waiting for CAMHS support suggests that the level of need far outweighs support, particularly for the most vulnerable or those in minority groups. In our study, it was the young people who appeared to be less likely to access services, suggesting services and policy-makers should prioritise outreach and support for this group [40].

Future studies should examine patterns of service contact throughout COVID-19 using data from the MHCYP 2017 follow-on survey series once available. It is also fundamental to understand how the proportions of children and young people reporting contact with DSM-V diagnosis actually equate to care given, for example the amount of therapeutic intervention received. Routine and linked data are likely to be helpful sources for this. Another of our key findings was the lower levels of contact amongst children and young people from minority ethnic backgrounds and young people in general, which is counter to early intervention [2, 3]. Further work needs to be done to address barriers to mental health service access by different ethnic groups, including the factors which may affect recognition of problems, and the impact of the format and delivery of service provision [14, 25, 40–42]. Improved recruitment, representation and inclusion in study design and samples are also crucial. Small samples and broader categories can mean results lack nuance or obscure differences between ethnic and cultural groups, and do not enable us to explore the intersectionality between groups [28, 40].

## Conclusion

There is increasing pressure on services to deliver mental health support following COVID-19. This paper provides a representative benchmark and prevalence of professional service use reported by parents of children and young people prior to the pandemic, and continuity with previous national surveys. Future data access to MHCYP follow-up surveys are vital to target specific services and monitor unmet need to improve funding and shape services and support.

### Supplementary Information

Below is the link to the electronic supplementary material.

Supplementary file1 (DOCX 35 KB)

## Data Availability

MHCYP 2017 survey data is available through data access request to NHS England’s DARS service, data.applications@nhsdigital.nhs.uk
