# Supplementary material for: Children and young people’s reported contact with professional services for mental health concerns: a secondary data analysis
Source: Eur Child Adolesc Psychiatry. 2024 Jan 4;33(8):2647–55. doi: 10.1007/s00787-023-02328-z (PMC11272805; doi:10.1007/s00787-023-02328-z)
Supplement: Supplementary file 1 — Supplementary file1 (DOCX 35 KB) [file 787_2023_2328_MOESM1_ESM.docx]

**Children and Young People’s Reported Contact with Professional Services for Mental Health Concerns: A Secondary Data Analysis**

European Child & Adolescent Psychiatry

**Frances Mathews^1*^**, Tamsin Jane Ford^2^, Simon White^3^, Obioha Chukwunyere Ukoumunne^4^, Tamsin Newlove-Delgado^1^

***Corresponding author email**

f.mathews2@exeter.ac.uk

**Affiliations**

1 University of Exeter Medical School

2 School of Clinical Medicine, Department of Psychiatry, University of Cambridge

3 MRC Biostatistics Unit, University of Cambridge, UK

4 NIHR Applied Research Collaboration South West Peninsula, Department of Health and Community Sciences, Faculty of Health and Life Sciences, University of Exeter

**Table 1 Participant characteristics of those having contact with a professional service for mental health concerns**

| **Sample characteristics** | **Total sample (N)** | **Proportion of sample reporting Professional contact %** |
| --- | --- | --- |
| **Age** |  |  |
| 5-10 | 3583 | 20.5 |
| 11-16 | 3098 | 21.5 |
| 17-19 | 927 | 22.0 |
|  |  |  |
| **Gender** |  |  |
| Female | 3,784 | 20.0 |
| Male | 3,824 | 22.2 |
|  |  |  |
| **Ethnicity** |  |  |
| White British / Other | 6,053 | 23.5 |
| Black / African / Caribbean / Black British | 312 | 9.6 |
| Asian / Asian British | 774 | 10.1 |
| Mixed / Multiple / Other | 467 | 16.1 |
|  |  |  |

**Table 2a: Prevalence and 95% confidence intervals (CI) for contact with services in children aged 5 to 10 as reported by parents, by gender and mental health disorder status**

| **Professional Service type** | | | **Any Professional Service** | **Teacher and School Staff** | **Primary Health Care** | **Mental Health Specialist** |
| --- | --- | --- | --- | --- | --- | --- |
| **DSM-IV disorder** | **Gender** | **N** | *Percentage (95%CI*)* | | | |
| **Any DSM-IV** | ***f*** | 145 | 56.7  (48.1-64.9) | 47.2  (38.8-55.7) | 30.1  (25.6-41.5) | 13.8  (8.9-20.8) |
|  | ***m*** | 285 | 66.7  (60.8-72.2) | 55.5  (49.4-61.4) | 33.7  (28.3-39.7) | 16.3  (12.2-21.3) |
| **Any anxiety disorder** | ***f*** | 55 | 63.4  (49.5-75.3) | 57.2  (43.5-69.9) | 39.8  (27.4-53.7) | 18.5  (9.9-31.9) |
|  | ***m*** | 75 | 69.3  (57.6-78.9) | 61.7  (49.9-72.3) | 33.2  (23.1-45.2) | 19.7  (11.9-30.9) |
| **Any depressive disorder** | ***f*** | 10 | 62.8  (20.5-91.7) | 48.8  (13.4-85.5) | 28.4  (4.7-76.2) | 15.9  (1.2-73.9) |
|  | ***m*** | 15 | 94.5  (62.4-99.4) | 79.7  (47.0-94.6) | 51.7  (23.6-78.7) | 35.8  (13.3-66.9) |
| **Any behavioural disorder** | ***f*** | 50 | 80.8  (66.1-90.0) | 63.8  (48.5-76.7) | 49.3  (34.8-63.9) | 17.3  (8.6-32.0) |
|  | ***m*** | 110 | 83.4  (75.1-89.4) | 71.4  (62.0-79.3) | 49.9  (40.4-59.5) | 25.2  (17.7-34.5) |
| **ADHD** | ***f*** | 25 | 88.9  (68.8-96.7) | 80.3  (58.7-92.1) | 60.1  (38.9-78.1) | 23.4  (10.0-45.6) |
|  | ***m*** | 90 | 85.5  (74.6-92.2) | 71.6  (60.5-80.6) | 48.4  (37.6-59.3) | 26.8  (18.1-37.7) |
| **ASD** | ***f*** | 10 | 100 | 88.4  (33.7-99.1) | 87.0  (30.8-99.0) | 32.7  (6.9-75.9) |
|  | ***m*** | 40 | 90.2  (75.5-96.5) | 90.2  (75.5-96.5) | 50.5  (34.3-66.7) | 34.6  (20.6-51.9) |
| **More than one disorder** | ***f*** | 35 | 94.2  (78.0-98.7) | 82.0  (64.2-92.0) | 72.1  (54.4-84.9) | 31.3  (17.6-49.4) |
|  | ***m*** | 95 | 85.5  (76.6-91.4) | 72.7  (62.6-80.9) | 48.1  (37.9-58.5) | 32.6  (23.5-43.1) |

***F=female; m=male; *CI=confidence interval***

**Table 2b: Prevalence and 95% confidence intervals (CI) for contact with services as reported by parents for children aged 11 to 16 years by gender and mental health disorder status**

| **Professional service type** | | | **Any Professional Service** | **Teacher and School Staff** | **Primary Health Care** | **Mental Health Specialist** |
| --- | --- | --- | --- | --- | --- | --- |
| **DSM-IV disorder** | **Gender** | **n** | *Percentage (95% CI)* | | | |
| **Any DSM-IV** | ***f*** | 230 | 61.5  (54.5-68.1) | 46.3  (39.6-53.2) | 29.5  (23.7-36.1) | 25.9  (20.4-32.3) |
|  | ***m*** | 265 | 66.0  (59.8-71.7 | 52.9  (46.6-59.1) | 29.9  (24.4-35.9) | 22.4  (17.6-28.0) |
| **Any anxiety disorder** | ***f*** | 145 | 60.8  (52.0-69.0) | 45.7  (37.3-54.3) | 32.1  (24.7-40.5) | 31.2  (23.9-39.6) |
|  | ***m*** | 95 | 75.1  (64.4-83.4) | 62.4  (51.5-72.2) | 43.4  (33.1-54.2) | 30.8  (21.8-41.5) |
| **Any depressive disorder** | ***f*** | 65 | 72.6  (59.5-82.7) | 54.4  (41.4-66.9) | 33.2  (22.4-46.1) | 30.3  (19.9-43.2) |
|  | ***m*** | 30 | 68.2  (48.5-83.1) | 52.2  (33.8-70.0) | 40.8  (24.1-59.8) | 25.7  (13.0-44.5) |
| **Any behavioural disorder** | ***f*** | 75 | 78.0  (66.8-86.2) | 55.0  (43.2-66.2) | 38.0  (27.4-49.8) | 38.9  (28.2-50.8) |
|  | ***m*** | 100 | 79.5  (69.6-86.7) | 59.9  (49.4-69.4) | 38.1  (28.8-48.3) | 31.3  (22.6-41.5) |
| **ADHD** | ***f*** | 25 | 83.9  (58.3-95.1) | 75.8  (51.3-90.3) | 42.5  (22.9-64.9) | 32.7  (15.7-56.0) |
|  | ***m*** | 80 | 83.1  (72.0-90.4) | 61.9  (50.1-72.4) | 37.0  (26.7-48.3) | 35.5  (25.4-47.1) |
| **ASD** | ***f*** | 10 | 86.1  (49.8-97.5) | 71.4  (36.8-91.5) | 56.9  (25.1-83.9) | 38.6  (12.9-72.7) |
|  | ***m*** | 25 | 82.5  (61.3-93.4) | 74.0  (53.2-87.8) | 25.1  (11.5-46.3) | 25.2  (11.5-46.6 |
| **More than one disorder** | ***f*** | 95 | 79.9  (69.8-87.2) | 58.8  (48.0-68.8) | 37.8  (28.1-48.5) | 38.6  (28.9-49.4) |
|  | ***m*** | 105 | 81.9  (72.3-88.7) | 60.4  (50.1-69.9) | 42.7  (33.1-52.9) | 32.9  (24.2-43.0) |

***f=female; m=male; *CI=confidence interval***

**Table 3c: Prevalence and 95% confidence intervals (CI) for contact with services as reported by parents for children aged 17 to 19 years by gender and mental health disorder status**

| **Professional service type** | | | **Any Professional Service** | **Teacher and School Staff** | **Primary Health Care** | **Mental Health Specialist** |
| --- | --- | --- | --- | --- | --- | --- |
|  | **Gender** | **n** | *Percentage (95% CI)* | | | |
| **Any DSM-V disorder** | ***f*** | 110 | 58.2  (48.1-67.8) | 30.1  (21.6-40.3) | 25.5  (17.9-34.8) | 28.9  (20.8-38.5) |
|  | ***m*** | 70 | 37.8  (26.7-50.4) | 18.0  (10.4-29.5) | 18.3  (10.7-29.5) | 19.4  (11.4-30.8) |
| **Any anxiety disorder** | ***f*** | 90 | 55.1  ( 44.0-65.7) | 27.3  (18.5-38.2) | 25.9  (17.6-36.3) | 26.7  (18.3-37.3) |
|  | ***m*** | 35 | 47.5  (30.1-65.5) | 26.8  (13.4-46.4) | 23.9  (12.0-41.9) | 25.4  (12.6-44.7) |
| **Any depressive disorder** | ***f*** | 35 | 73.1  (53.5-86.5) | 34.1  (18.7-53.7) | 45.6  (28.2-64.1) | 45.4  (28.1-63.9) |
|  | ***m*** | 15 | 51.0  (25.2-76.3) | 27.1  (9.3-57.4) | 38.4  (16.2-66.7) | 19.0  (5.4-49.3) |
| **Any behavioural disorder** | ***f*** | 5 | - | - | - | - |
|  | ***m*** | 5 | 29.2  (3.2-83.8) | - | 10.0  (0.5-70.8) | 19.3  (1.1-84.1) |
| **ADHD** | ***f*** | - |  | - | - | - |
|  | ***m*** | 15 | 35.1  (13.4-65.4) | 15.2  (3.7-43.4) | 4.9  (0.5-34.9) | 6.9  (0.7-43.4) |
| **ASD** | ***f*** | - | - | - | - | - |
|  | ***m*** | 5 | 27.6  (2.2-86.6) | 13.6  (0.5-83.7) | 14.0  (0.5-84.2) | 13.6  (0.5-83.7) |
| **More than one disorder** | ***f*** | 30 | 73.7  (52.2-87.8) | 30.3  (14.6-52.7) | 38.5  (21.3-59.1) | 43.3  (25.0-63.6) |
|  | ***m*** | 15 | 42.7  (19.9-69.2) | 25.2  (8.5-55.1) | 25.9  (9.3-54.3) | 13.2  (2.8-44.2) |

***f=female; m=male; *CI=confidence interval***
